# Analysis of the In Situ Crack Evolution Behavior in a Solid Solution Mg-13Gd-5Y-3Zn-0.3Zr Alloy

**DOI:** 10.3390/ma14010036

**Published:** 2020-12-24

**Authors:** Yaqin Yang, Chongli Mu, Zhongjian Han, Jian Xu, Baocheng Li

**Affiliations:** College of Materials Science and Engineering, North University of China, Taiyuan 030051, China; muchongli@163.com (C.M.); hzj4965@163.com (Z.H.); xujian_8726@sina.com (J.X.); libaocheng@nuc.edu.cn (B.L.)

**Keywords:** Mg-13Gd-5Y-3Zn-0.3Zr alloy, solid solution treatment, in situ tensile test, micro-cracks, LPSO phase, the LPSO/α-Mg interface, the grain boundary

## Abstract

The low plasticity of high strength Mg-Gd-Y alloy has become the main obstacle to its application in engineering. In this paper, the origin, propagation and fracture processes of cracks of a solution of treated Mg-13Gd-5Y-3Zn-0.3Zr alloy were observed and studied with scanning electron microscopy (SEM) in an in situ tensile test to provide theoretical references for the development of a new high-performance Mg-Gd-Y alloy. The results showed that there was still some bulk long period stacking order (LPSO) phase remaining in solid solution Mg-13Gd-5Y-3Zn-0.3Zr alloy. Most importantly, it was found that the locations of micro-cracks vary with the different solution treatment processes, mainly including the following three types. (1) At 480 × 10 h and 510 °C × 10 h, much bulk LPSO phase with higher elastic modulus remains in the alloy, which can lead to micro-cracks in the LPSO phase due to stress concentration. (2) At 510 °C × 13 h and 510 °C × 16 h, the phase structure of bulk LPSO changes, and the stress concentration easily appears at the LPSO/α-Mg interface, which leads to micro-cracks at the interface. (3) At 510 °C × 19 h and 510 °C × 22 h, the grain size increases, and the stress concentration is obvious at the grain boundary of coarse grains, which leads to the formation of micro-cracks.

## 1. Introduction

Magnesium alloys are mainly used in aerospace, weapon equipment, automobile shell and other fields due to several advantages, including low density, high specific strength and good thermal and electrical conductivity. The research and development of high-performance wrought rare earth magnesium alloys has become more prominent in recent years. Adding small amounts of rare earth elements (RE) is a common method of increasing the strength of magnesium alloys. It has led to the development of Mg-Nd, Mg-Y, Mg-Gd and Mg-Gd-Y-Zr rare earth magnesium alloys [1,2]. Due to the high solid solubility of rare earth elements in the magnesium matrix and precipitation strengthening, a Mg-RE magnesium alloy (RE/Zn weight ratio >1, RE = Y, Gd, Tb, Dy, Ho, Er, Tm) has wider application prospects in the field of high-strength and heat-resistant industry. High-strength Mg-RE alloys generally contain Gd and Y as alloying elements. Mg-Gd-Y alloys not only have high strength and toughness, but also high corrosion resistance and good mechanical properties at high temperatures [3,4,5]. As the proportion of rare earth elements in the alloy increases, the room temperature strength and high temperature heat resistance of the alloy increase, but the plastic deformation ability decreases. Due to the closely packed hexagonal structure of magnesium alloy, it has a small number of slip systems, which make its plastic deformation behavior difficult [6,7]. It is also easy to cause cracks during deformation due to poor plasticity. Therefore, it is necessary to study the deformation behavior and fracture mechanisms of magnesium alloys.

In most current research, fracture behavior has usually been based on speculation of the fracture process by observing fractographies following tensile tests. The in situ tensile test is mainly used for deformation or fracture micro zone analysis. This is used to observe the crack process in real time and is very useful in the study of material fracture mechanisms. It is a powerful technique that can investigate the details of microstructural evolution, deformation behavior and fracture mechanisms during the deformation process. Wang et al. [8] carried out in situ tensile tests with a SEM and found that small cracks are formed preferentially on the boundary of the α-Mg_17_Al_12_ phase, and crack growth only occurs when the crack length is less than 1 mm in cast AM50 and AM60B magnesium aluminum alloys. Cui et al. [9] studied the crack evolution behavior of a Mg-8.07Al-0.53Zn-1.36Nd magnesium alloy and found that cracks are mainly formed in the interior of the phase, propagating in two ways (intergranular and transgranular). However, there are very few studies regarding in situ observations of the dynamic fracture process and microstructural evolution on Mg-RE alloy. Most Mg-RE alloys exhibit excellent normal and high temperature yield strength, excellent elongation and higher strain rate superplasticity due to the special LPSO phase structure. The LPSO phase is a long-period structure phase produced by the long-range orderly stacking of close-packed layers, and it is a lamellar precipitation phase precipitated from the base surface of the magnesium matrix. At present, the research on LPSO phase mainly focuses on the structural characteristics, formation mechanism, strengthening and toughening effects on the matrix, mutual transformation and development of new alloy systems [10,11,12,13,14]. This paper consists of an in-depth analysis of crack initiation and propagation behavior in solid solution Mg-13Gd-5Y-3Zn-0.3Zr alloy containing LPSO phase, using SEM and an in situ loading method. This provides a theoretical basis for the optimization process and the safe use of magnesium alloys, and a reference for the development of new high-performance magnesium alloys.

## 2. Materials and Experimental Methods

Upsetting and drawing deformed Mg-13Gd-5Y-3Zn-0.3Zr alloy was prepared for research. The material was provided by the Precision Forming Center of North China University, Shanxi. The alloy has excellent mechanical properties at room temperature and high temperature, and can be used for some high-mechanical-property components in aerospace, railway transportation and other fields. The density value of the alloy is 2.02 g/cm^3^ and the chemical composition (wt%) of the alloy is listed in Table 1.

Zhang et al. [15] found that there are still some second phases in a Mg-13Gd-5Y-3Zn-0.3Zr alloy after solid solution. This is mainly due to the high content of alloying elements. It was also found that with solution temperatures of 480 and 510 °C, the amounts of second phases in the alloy were fewer and the degree of solid solution was better. In this paper, solid solution treatments of 480 °C × 10 h, 510 °C × 10 h, 510 °C × 13 h, 510 °C × 16 h, 510 °C × 19 h and 510 °C × 22 h were selected for investigation. The cooling method used was water cooling, with a water temperature of about 70 °C.

**Figure 1:**
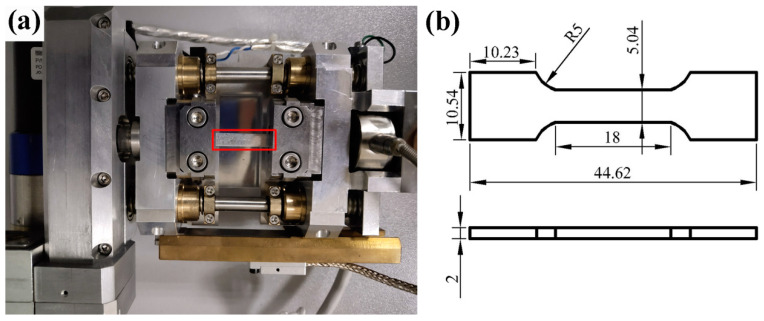
(**a**) In situ tensile testing system. (**b**) The machined size of the in situ tensile specimen (unit: mm).

To prepare, grind and polish the sample, and corrode the surface with 1 g picric acid, 2.5 mL glacial acetic acid, 2.5 mL absolute ethanol and 18 mL distilled water. A Neophot2 optical microscope (OM, Zeiss, Oberkochen, Germany) and SU-5000 scanning electron microscope (SEM, Hitachi SU5000, Tokyo, Japan) were used to analyses the microstructure of the deformed and the solution-treated samples. The grain size was measured by the cut-line method. The compositions of the phases and compounds in the alloy were analyzed using energy dispersive spectroscopy (EDS, EDAX Inc., Mahwah, NJ, USA). A DX-2700 x-ray diffraction analyzer (XRD, DX-2700, Dandong, China) was used to determine the phase of the sample. The parameters used were scanning speed 5 °/min, scanning step 0.05°, scanning range 20–80°, acceleration voltage 40 KV and filament current 30 mA. The in situ tensile test was developed using an SU-5000 SEM (SEM, Hitachi SU5000, Tokyo, Japan). The supporting in situ tensile stage is shown in Figure 1a and the machined size is shown in Figure 1b. The preparation steps of in situ tensile test specimen were as follows: Firstly, the observed surface was roughened with coarse sandpaper, and then the hot-melt wax was heated to make the back of the tensile sample stick to the metal block. Then the tensile specimens were prepared according to the OM specimen preparation method. After chemical corrosion, the hot-melt wax was reheated to soften it, and then the sample was removed; the residual hot-melt wax on the surface of tensile sample was cleaned with acetone and alcohol.

The tensile test rate was 0.5 mm/min. Before the sample yields, the SEM picture was captured once every 0.5 mm of the fixture displacement. After yielding, the picture was captured every 0.2 mm. During the tensile test, as soon as a crack was observed, the loading was stopped and the corresponding stress and strain were recorded.

## 3. Results

### 3.1. Microstructure Analysis

**Figure 2:**
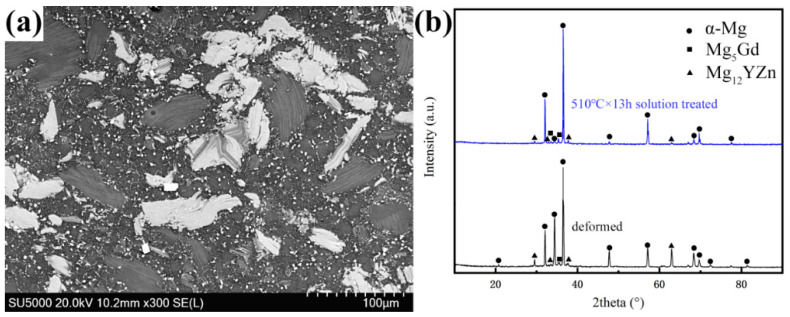
SEM photo and XRD results. (**a**) SEM photo of the deformed alloy. (**b**) XRD results of the deformed and 510 °C × 13 h solution-treated alloys.

Figure 2a is the SEM image of the deformed sample. Figure 2b shows the XRD results of the deformed and solution-treated samples. It can be seen from Figure 2a that there are many second phases in the deformed alloy, mainly including four types: dispersed white punctate phase, gray bulk phase, lamellar phase and a small amount of white cubic-shaped phase. It can be seen from Figure 2b that the phase composition includes α-Mg, Mg_12_YZn and Mg_5_Gd phases in the deformed alloy. After 510 °C × 13 h solution treatment, the phase composition mainly includes the α-Mg and Mg_12_YZn; some diffraction peaks of Mg_5_Gd phase disappear because of the dissolution of Mg_5_Gd phase in the matrix.

**Figure 3:**
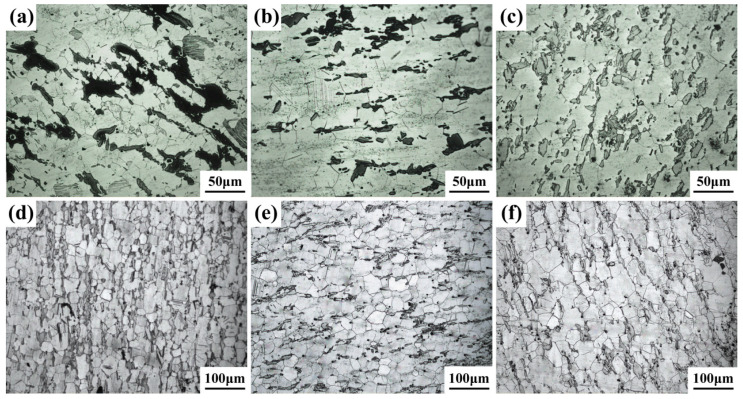
Optical microscopy OM photos of the alloy after different solid solution treatments. (**a**) 480 °C × 10 h. (**b**) 510 °C × 10 h. (**c**) 510 °C × 13 h. (**d**) 510 °C × 16 h. (**e**) 510 °C × 19 h. (**f**) 510 °C × 22 h.

Figure 3 shows the OM photos of the alloys after different solid solution treatments. When the solid solution is at 480 °C for 10 h, bulk phases can obviously be seen on the grain boundaries, as shown in Figure 3a. When the solid solution was at 510 °C for 10 or 13 h, the volume fraction of the bulk phase decreased and the grain size was slightly larger than that at 480 °C, as shown in Figure 3b,c. Additionally, at 510 °C for 16, 19 and 22 h, it can be seen that with an increase in heating time, some bulk phases gradually refine and decompose, distributing around grain boundaries in the form of dots or stripes, and the grain size increases. Table 2 shows the grain size of Mg-13Gd-5Y-3Zn-0.3Zr alloys after different solution treatments. It was found that the grain size increases with increases of temperature and heating time.

**Figure 4:**
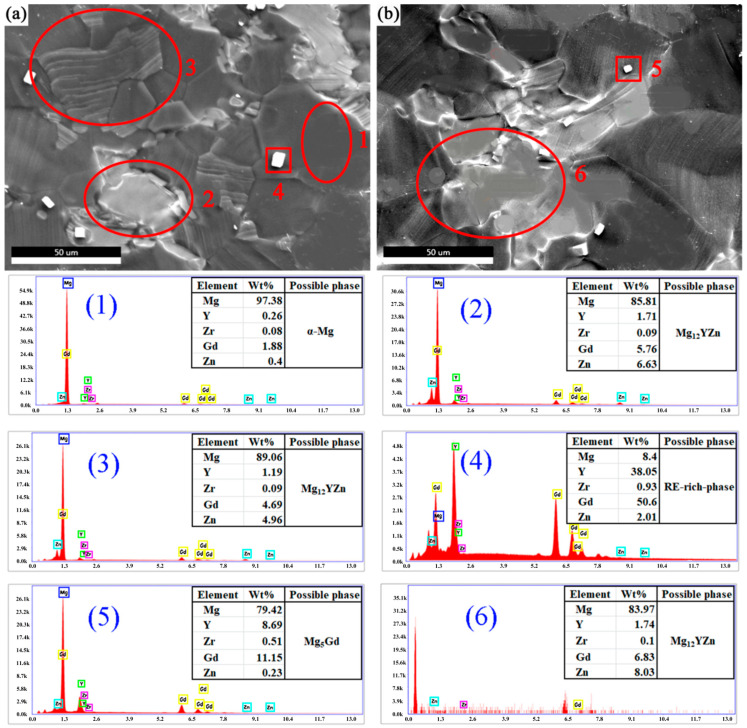
EDS analysis selection map of 510 °C × 13 h solid solution alloy. (**a**,**b**) correspond to different positions on the same sample.

Take the sample of 510 °C × 13 h as an example. Figure 4 shows the results of EDS analysis. Figure 4a,b comes from the same sample. The atomic ratio of the Mg element at spot (1) in Figure 4 is close to 100%, indicating that it is the α-Mg matrix phase. The chemical composition of the block-shaped phase (Figure 4, spot (2)) was Mg-5.76Gd-1.71Y-6.63Zn-0.09Zr (at.%), which indicates that the stoichiometry of this phase was near Mg_12_YZn (LPSO phase). The chemical composition of the lamellar phase (Figure 4, spot (3)) was Mg-4.69Gd-1.19Y-4.96Zn-0.09Zr (at.%). Furthermore, the lamellar phase emerged inside α-Mg grains and the content of Mg was high due to the selected part of the matrix phase. Though the morphologies of the phases were different, spot (2) and spot (3) were considered to be the same phase due to their similar element content. This may be the LPSO phase. The LPSO phase is an ordered structure in which the main components are RE and Zn atoms placed periodically in the Mg basal planes [16,17]. As an important type of Mg alloy, Mg-RE-Zn alloys have attracted attention recently due to their enhanced properties caused by their LPSO structure. The cubic-shaped phase (Figure 4, spot (4)) could be considered to be an RE-rich compound due to the EDS results. The chemical composition of the phase (Figure 4, spot (5)) was Mg-11.15Gd-8.69Y-0.23Zn-0.51Zr (at.%), which indicates that the stoichiometry of this phase is near Mg_5_Gd. However, the Mg content was high due to the selected part of the matrix phase. The chemical composition of the phase (Figure 4, spot (6)) was Mg-6.83Gd-1.74Y-8.03Zn-0.1Zr (at.%), which indicates that the stoichiometry of this phase was near Mg_12_YZn. Basically, according to the XRD and EDS results, the black phase was the α-Mg matrix phase. The granular phase was mainly Mg_5_Gd. The main component of the grey layer LPSO phase and the bulk LPSO phase was Mg_12_YZn. The white square phase was the RE-rich phase.

### 3.2. Dynamic Crack Initiating and Extending Behavior

**Figure 5:**
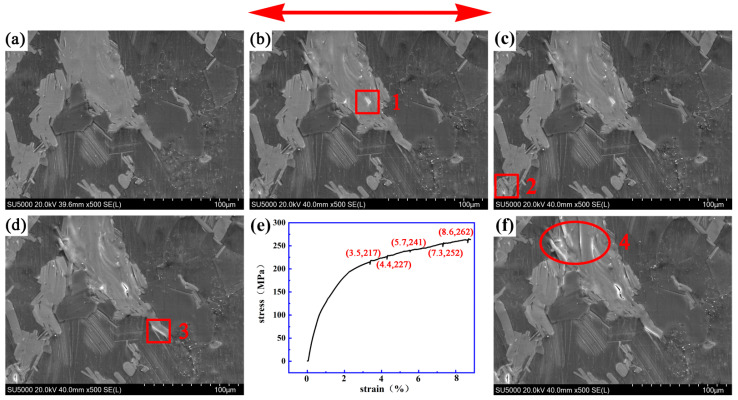
The dynamic process of crack initiating and extending in 510 °C × 13 h solid solution Mg-13Gd-5Y-3Zn-0.3Zr alloy. (**a**) ε = 0, σ = 0 MPa. (**b**) ε = 3.5%, σ = 217 MPa. (**c**) ε = 4.4%, σ = 227 MPa. (**d**) ε = 5.7%, σ = 241 MPa. (**e**) The stress–strain curve. (**f**) ε = 7.3%, σ = 252 MPa.

Figure 5 shows the dynamic process of 510 °C × 13 h solid solution Mg-13Gd-5Y-3Zn-0.3 Zr alloy. Figure 5e shows the stress–strain curve, and the corresponding stress and strain of the new crack that appeared during the in situ tension process have been marked. The stress drop on the curve was caused by pausing the loading stress when the crack picture was captured. Figure 5a shows the original microstructure before loading. It mainly includes the α-Mg matrix phase and the bulk LPSO phase. When the strain and stress increased from a strain value of 0 and a stress value of 0 MPa to a strain value of 3.5% and a stress value of 217 MPa, a small crack “1” appeared in the bulk LPSO phase of about 6.5 μm in length, as shown in Figure 5a,b. When the strain and stress increased to a strain value of 4.4% and a stress value of 227 MPa, a small hole “2” appeared in the bulk LPSO phase and became a new crack source. At this point, the length of crack “1” increased to 7.8 μm and slightly widened, as shown in Figure 5c. When the strain and stress increased to a strain value of 5.7% and a stress value of 241 MPa, a new crack “3” appeared at the LPSO/α-Mg interface. At this point, there was a slight change to crack “1” and the length of crack source “2” increased to about 5.2 μm, as shown in Figure 5d. When the strain and stress increased to a strain value of 7.3% and a stress value of 252 MPa, the length of crack “1” increased to 16.9 μm, crack “2” widened to about 6.5 μm and crack “3” extended to about 15.6 μm along the LPSO/α-Mg interface. White lines (similar to folding) can be seen inside the LPSO phase in the circle region. These may develop into new cracks, as shown in Figure 5f. Fracturing occurs when the strain and stress are 8.6% and 262 MPa, respectively. The initial length of crack “1” was 6.5 μm and the final length was 16.9 μm. The fracture stress of the specimen was 262 MPa.

## 4. Discussion

In a similar way, other groups of solution-treated samples were observed in situ. It was found that the locations of the micro-cracks differed with various solution treatment processes, including the following three types:

(1) When the solid solution treatment process was 480 °C for 10 h or 510 °C for 10 h, the micro-cracks mainly appeared in the bulk LPSO phase.

(2) When the solid solution treatment process was 510 °C × 13 h or 510 °C × 16 h, the micro-cracks appeared in the bulk LPSO phase and at the interface of the LPSO phase/α-Mg.

(3) When the solution treatment process was 510 °C × 19 h or 510 °C × 22 h, the micro-cracks mainly appeared at the grain boundary.

### 4.1. Initiation and Propagation Behaviur of Cracks in the Bulk LPSO Phase

**Figure 6:**
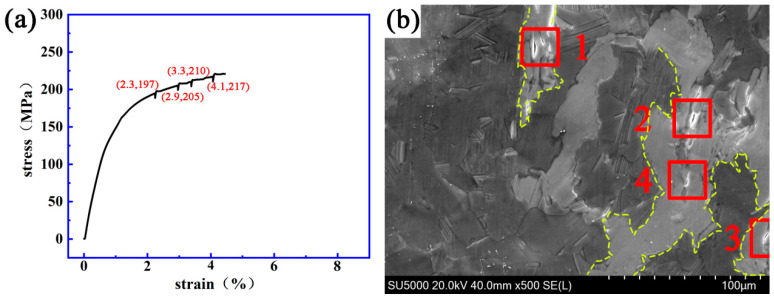
The dynamic change process of crack initiating and extending a Mg-13Gd-5Y-3Zn-0.3Zr alloy after solution treatment at 480 °C for 10 h. (**a**) The stress–strain curve. (**b**) The evolution process of the micro-crack.

**Figure 7:**
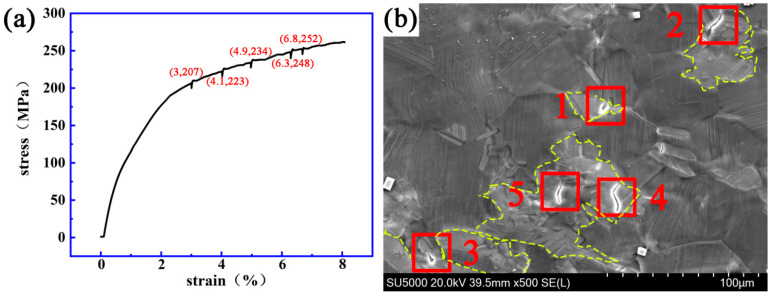
The dynamic change process of crack initiating and extending a Mg-13Gd-5Y-3Zn-0.3Zr alloy after solution treatment at 510 °C for 10 h. (**a**) The stress–strain curve. (**b**) The evolution process of the micro-crack.

Figure 6 and Figure 7 show the stress–strain curve and the dynamic micro-crack initiating and extending process observed in situ using a SEM on a Mg-13Gd-5Y-3Zn-0.3Zr alloy after solution treatment at 480 °C for 10 h and 510 °C for 10 h.

Figure 6 shows that when the strain is 2.3%, 2.9%, 3.3% or 4.1%, and the stress is 197, 205, 210 or 217 MPa, respectively, cracks “1”, “2”, “3” and “4” are initiated, respectively. The initial length of crack “1” was 1.2 μm and the final length was 9.1 μm. The fracture strain and stress of the specimen were 4.5% and 220 MPa. All four cracks appeared in the bulk LPSO phase.

Figure 7 shows that when the strain is 3%, 4.1%, 4.9%, 6.3% or 6.8%, respectively, and the stress is 207, 223, 234, 248 or 252 MPa, respectively, cracks “1”, “2”, “3”, “4” and “5” are initiated, respectively. The initial length of crack “1” was 0.9 μm and the final length was 5.2 μm. The fracture strain and stress of the specimen were 7.9% and 262 MPa. Among the five cracks, four appeared in the bulk LPSO phase (cracks “1”, “3”, “4” and “5”).

From the in situ tensile tests at 480 °C × 10 h and 510 °C × 10 h, it can be seen that the cracks mainly occur in the bulk LPSO phase, which indicates that the stress concentration is highest in this phase. LPSO/α-Mg two-phase alloy can be regarded as composite material approximately. LPSO phase and α-Mg phase are reinforcement phase and matrix phase respectively. Oňorbe et al. [18] also found similar experimental phenomena in their study of the strain evolution of a Mg-Y-Zn alloy containing an LPSO phase. First, a Mg_97_Y_2_Zn_1_Gd_0.5_ (at.%) alloy was prepared; then the Young’s modulus of the LPSO phase and the α-Mg matrix phase were measured using the micro-indentation method. The values were 75 and 48 GPA, respectively. In addition, the change in the elastic strain energy of the LPSO/α-Mg alloy during in situ tension was also measured. It was found that the stress of the α-Mg matrix gradually transfers to the LPSO phase, due to its elastic modulus being far higher than that of the α-Mg matrix phase under external loading. Therefore, the stress of the LPSO phase is gradually higher than the external stress, leading to the cracks in the LPSO phase.

### 4.2. Initiation and Propagation Behavior of the Cracks at the LPSO/α-Mg Interface

**Figure 8:**
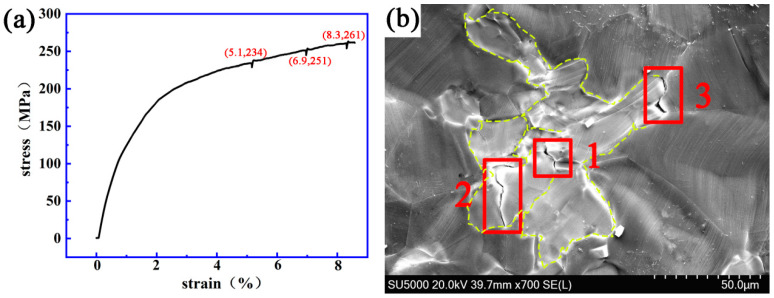
The dynamic change process of crack initiating and extending a Mg-13Gd-5Y-3Zn-0.3Zr alloy after solution treatment at 510 °C for 16 h. (**a**) The stress–strain curve. (**b**) The evolution process of the micro-crack.

Figure 8 shows the stress–strain curve and the dynamic micro-crack initiating and extending process observed in situ using a SEM on a Mg-13Gd-5Y-3Zn-0.3Zr alloy after solution treatment at 510 °C for 16 h.

This shows that when the strain is 5.1%, 6.9% and 8.3%, respectively, and the stress is 234 MPa, 251 MPa and 261 MPa, respectively, cracks “1”, “2” and “3” are initiated, respectively. The initial length of crack “1” is 1.4 μm and the final length is 3.8 μm. The fracture strain and stress of the specimen is 8.5% and 263 MPa. It was also found that in addition to crack initiation inside the bulk LPSO phase, cracks also appeared at the interface of LPSO/α-Mg.

According to the structure, the LPSO phase can be divided into R-type and H-type, including 6H, 10H, 15R, 14H, 18R and 24R. The 18R-LPSO phase is not stable, and the 18R-LPSO phase changes into the more stable 14H-LPSO phase at higher temperatures [19,20,21,22,23,24] Nishida et al. [25] found that the 18R-LPSO phase mainly existed around the grain boundary of the α-Mg matrix in as-cast Mg_97_Y_2_Zn_1_ alloy, and after heat treatment at 773k for 5 h, the structure of 18R-LPSPO gradually transformed into 14H-LPSO. Liu Huan [26] found that in the tensile process at room temperature, the blocking ability of the 14H-LPSO phase to dislocation motion is much greater than that of the 18R-LPSO phase.

When dislocations move to the interface between the α-Mg matrix and the 14H-LPSO phase, stress concentration will be formed. The 14H-LPSO phase has strong anisotropy and the deformation mode is single. When plastic deformation occurs, the 14H-LPSO phase is incoMPatible with the deformation frequency of the matrix, resulting in cracks at the interface of LPSO/α-Mg.

### 4.3. Initiation and Propagation Behavior of Cracks on the Grain Boundary

**Figure 9:**
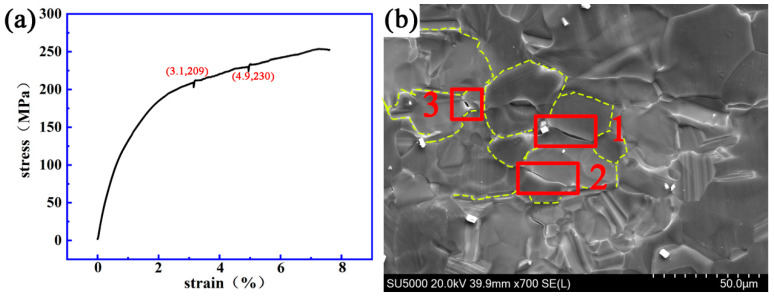
The dynamic change process of crack initiating and extending a Mg-13Gd-5Y-3Zn-0.3Zr alloy after solution treatment at 510 °C for 19 h. (**a**) The stress–strain curve. (**b**) The evolution process of the micro-crack.

**Figure 10:**
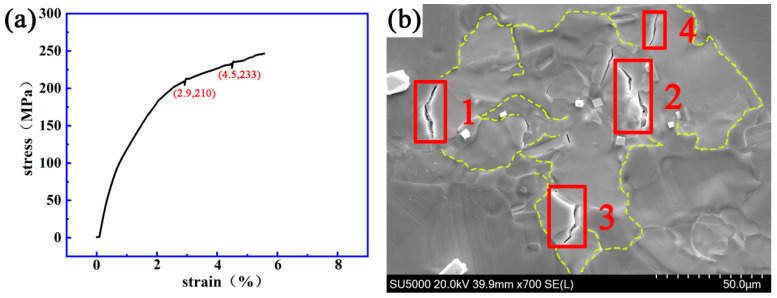
The dynamic change process of crack initiating and extending a Mg-13Gd-5Y-3Zn-0.3Zr alloy after solution treatment at 510 °C for 22 h. (**a**) The stress–strain curve. (**b**) The evolution process of the micro-crack.

Figure 9 and Figure 10 show the stress–strain curve and the dynamic micro-crack initiating and extending process observed in situ using a SEM on a Mg-13Gd-5Y-3Zn-0.3Zr alloy after solution treatment at 510 °C for 19 h and 510 °C for 22 h, respectively.

Figure 9 shows that when the strain and stress are 3.1% and 209 MPa, respectively, crack “1” will appear on the grain boundary. When the strain and stress are 4.9% and 230 MPa, respectively, crack “2” will appear at the grain boundary; then crack “3” will also appear. The initial length of crack “1” was 3.8 μm and the final length was 3.9 μm. The fracture strain and stress of the specimen were 7.7% and 253 MPa.

Figure 10 shows that when the strain and stress are 2.9% and 210 MPa, respectively, crack “1” will appear on the grain boundary. When the strain and stress are 4.5% and 233 MPa, respectively, crack “2” will appear on the grain boundary; then cracks “3” and “4” will also appear, and the white second phase can be seen near the crack. The initial length of crack “1” was 1.3 μm and the final length was 12.1 μm. The fracture strain and stress of the specimen were 5.6% and 251 MPa.

It can be seen from Table 2 that the grain size of the alloy after solution treatment at 510 °C × 19 h and 510 °C × 22 h is about twice as large as that after solution treatment at 480 °C × 10 h. The plasticity of coarse grain material is worse than that of fine grain material. The crack initiation mechanism of the dislocation blockage can be used to explain the effect of grain size on the fracture behavior of the alloy. The mechanism was first proposed by Zener [27,28] in 1948, and then developed and perfected by Stroh et al. [29]. According to the dislocation stacking theory, the number of dislocations is mainly related to the slitting stress of the external force in the slip direction and the distance from the source of the dislocation to the obstacle, as shown in Equation (1) [30].

(1) $$n=\frac{k\pi \tau d}{Gb}$$

where “*n*” is the number of dislocations. “*G*” is the shear modulus of elasticity. “*k*” is the coefficient related to the type of dislocation: if the dislocation type is edge dislocation, *k* = (1 − μ); for screw dislocation, *k* = 1. “*τ*” is shear stress of external force in slip direction. “*d*” is the slip distance before the dislocation meets the obstruction (can be approximately regarded as the dislocation stacking group length or the average grain diameter) and “*b*” is Burgers vector.

When the coefficient “*k*”, shear stress “*τ*” and Burgers vector “*b*” are constant, the number of dislocations is proportional to the slip distance “*d*”. The larger the grain size, the longer the slip distance and the higher the dislocation density. Under external force, the dislocation source starts to move and the subsequent dislocations push it forward. When the leading dislocation encounters the barrier of the grain boundary, it will stop moving. The hindered leading dislocation has a repulsive force on the latter dislocation, which makes the latter dislocation stagnate. However, the whole stacking group of dislocations has a reaction force on the leading dislocation. The longer the dislocation slip distance and the more dislocations there are in the packing group, the greater the reaction force on the leading dislocation. This can be several times the external stress. When the stress concentration caused by dislocation stacking exceeds the strength of the grain boundary, the grain boundary will crack. According to Equation (1), under the same stress, the number of dislocations around the boundary of coarse grains is greater than in fine grains. Therefore, the stress concentration near the coarse grain boundary is more serious. The serious stress concentration can more easily initiate cracks.

He [31] studied the effects of heat treatment and alloy composition on the formation and propagation of cracks in cast Mg-Gd-Y-Zr(Ca) alloy. In the study, the crack initiation mechanism of dislocation blockage was used to explain the origin of the crack in the alloy. Through model modification and formula derivation, it was concluded that reducing the grain size can reduce the degree of stress concentration.

## 5. Conclusions

The fracture behavior of a solid solution high-strength Mg-13Gd-5Y-3Zn-0.3Zr alloy was analyzed in real time by observing the microstructural evolution using in situ SEM tensile testing. The fracture mechanisms were deduced, and the following conclusions were drawn.

(1) The phase composition includes α-Mg, Mg_12_YZn and Mg_5_Gd in the deformed alloy. After solid solution, bulk Mg_12_YZn (LPSO) phase still exists.

(2) When the solid solution treatment process is 480 °C for 10 h or 510 °C for 10 h, many LPSO phases remain in the alloy. These LPSO phases have higher elastic moduli and are prone to stress concentration, which leads to the brittle and straight micro-cracks in the LPSO phase.

(3) When the solid solution treatment process is 510 °C × 13 h or 510 °C × 16 h, the structure of bulk LPSO phase changes; the stress concentration occurs at the LPSO/α-Mg interface, resulting in micro-cracks appearing in the bulk LPSO phase and at the interface of the LPSO phase/α-Mg.

(4) When the solution treatment process is 510 °C × 19 h or 510 °C × 22 h, the grain size increases. The stress concentration at the grain boundary of coarse grains is serious, which leads to microcracks.

## Figures and Tables

**Table 1:** Chemical composition (wt%) of a Mg-13Gd-5Y-3Zn-0.3Zr alloy.

Mg	Gd	Y	Zn	Zr
Bal	12.77	4.5	2.65	0.34

**Table 2:** Grain size of a Mg-13Gd-5Y-3Zn-0.3Zr alloy after different solution treatment.

Solution Treatment	480 °C × 10 h	510 °C × 10 h	510 °C × 13 h	510 °C × 16 h	510 °C × 19 h	510 °C × 22 h
Grain size (µm)	17	24	26	31	32	34

## Data Availability

Data is contained within the article.
